# Supplementary material for: In Vitro and In Silico Anti-Arboviral Activities of Dihalogenated Phenolic Derivates of L-Tyrosine
Source: Molecules. 2021 Jun 5;26(11):3430. doi: 10.3390/molecules26113430 (PMC8201234; doi:10.3390/molecules26113430)
Supplement: Supplementary file 1 [file molecules-26-03430-s001.zip › Table S1 (06-01-2021).pdf]

Table S1. *In vitro* Viability of dihalogenated compounds derived from L-Tyrosine in U937 cells.

	Concentration (μM)	Viability Percentage	EEM
Ribavirin	100	87.9	4.25
Doxycycline	50	91.3	4.03
TDC	250	89.6	1.20
TDB	250	86.3	1.13
TDC_2M_ME	250	89.5	2.85
TDB_2M_ME	250	78.7	3.62
TDC_2M	250	94.0	3.96
TDB_2M	250	87.4	4.12
TDC_3M_ME	250	87.6	4.05
TDB_3M_ME	250	86.3	3.80
TDC_3M	250	91.2	2.86
TDB_3M	250	82.2	2.68
