# Supplementary material for: In Vitro and In Silico Anti-Arboviral Activities of Dihalogenated Phenolic Derivates of L-Tyrosine
Source: Molecules. 2021 Jun 5;26(11):3430. doi: 10.3390/molecules26113430 (PMC8201234; doi:10.3390/molecules26113430)
Supplement: Supplementary file 1 [file molecules-26-03430-s001.zip › Table S2 (06-01-2021).pdf]

Table S2: PFU/ml obtained with the treatment with di-halogenated compounds derived from L-Tyrosine in combined strategy.

	**DENV-2/S16803**	**ZIKV/Col**	**CHIKV/Col**
Control	$1.25x10^{06}$	$1.71x10^{06}$	$3.35x10^{17}$
Suramine	$<1.00x10^{00}$	$<1.00x10^{00}$	$<1.00x10^{00}$
Ribavirin	$6.25x10^{04}$	$9.80x10^{04}$	$2.63x10^{16}$
TDC	$7.69x10^{06}$	$4.63x10^{06}$	$1.49x10^{17}$
TDB	$6.00x10^{06}$	$4.44x10^{06}$	$1.13x10^{17}$
TDC_2M_ME	$3.25x10^{06}$	$5.85x10^{05}$	$7.63x10^{16}$
TDB_2M_ME	$2.94x10^{06}$	$9.27x10^{05}$	$5.06x10^{16}$
TDC_2M	$3.50x10^{06}$	$4.50x10^{06}$	$9.56x10^{16}$
TDB_2M	$1.83x10^{06}$	$4.69x10^{06}$	$1.16x10^{17}$
TDC_3M_ME	$3.56x10^{06}$	$4.06x10^{06}$	$1.26x10^{17}$
TDB_3M_ME	$6.31x10^{06}$	$7.44x10^{06}$	$1.70x10^{17}$
TDC_3M	$5.19x10^{06}$	$8.13x10^{06}$	$1.46x10^{17}$
TDB_3M	$3.69x10^{06}$	$3.94x10^{06}$	$1.34x10^{17}$
