# Supplementary material for: In Vitro and In Silico Anti-Arboviral Activities of Dihalogenated Phenolic Derivates of L-Tyrosine
Source: Molecules. 2021 Jun 5;26(11):3430. doi: 10.3390/molecules26113430 (PMC8201234; doi:10.3390/molecules26113430)
Supplement: Supplementary file 1 [file molecules-26-03430-s001.zip › Table S3 (06-01-2021).pdf]

Table S3: Estimated selectivity index of di-halogenated compounds derived from L-tyrosine.

	ZIKV/Col	CHIKV/Col
TDC	--	7.3
TDB	--	>1346.0
TDC_2M_ME	11.2	13.1
TDB_2M_ME	11.4	21.1
TDC_2M	--	136.2
TDB_2M	--	15.6
TDC_3M_ME	--	102.2
TDB_3M_ME	--	23.6
TDC_3M	--	7.1
TDB_3M	--	36.8
