# Supplementary material for: In Vitro and In Silico Anti-Arboviral Activities of Dihalogenated Phenolic Derivates of L-Tyrosine
Source: Molecules. 2021 Jun 5;26(11):3430. doi: 10.3390/molecules26113430 (PMC8201234; doi:10.3390/molecules26113430)
Supplement: Supplementary file 1 [file molecules-26-03430-s001.zip › Table S4 (05-11-2021).pdf]

Table S4: ***In silico*** **molecular interactions between TDC-2M-ME and TDB-2M-ME with viral and cellular proteins by LigPlot®.**

Virus. Protein and PDB code	Ligand	Free binding energy (Kcal/mol)	Hydrogen bonds	Residues and distance of H+ bonds (Å)	Hydrophobic interactions	Residues participating in hydrophobic interactions
ZIKV Envelope – Domain III (5JHM)	*TDC_2M_ME*	-4.80 ± 0.10	2	Ser146 (3.1); Lys373 (2.9)	20	Val143; Ser146; Gly145; Asn362; Pro363; Val364; Ile365; Thr366; Met374
	TDB_2M_ME	-4.70 ± 0.10	2	Val364 (2.8); Thr366 (3.0)	7	Ile1; Gly145; Ser146; Ile365; Lys373; Met374; Met375
ZIKV Envelope – Fusion Peptide (5JHM)	*TDC_2M_ME*	-5.00 ± 0.00	2	Cys3 (3.0); Gly5 (3.0)	7	Ile1; Arg2; Val6; Asp98; Arg99; Gly102; Asn103
	TDB_2M_ME	-4.87 ± 0.06	3	Cys3 (3.0; 3.5); Asp98 (3.1)	8	Ile1; Arg2; Ile4; Gly5; Val6; Arg99; Gly102; Asn103
ZIKV Core (5YGH)	*TDC_2M_ME*	-4.80 ± 0.00	2	Arg45 (3.3); Phe53 (3.3)	16	Arg45. Met46. Ala49. Ala52. Phe53. Phe56. Phe84. Asp87
	TDB_2M_ME	-4.67 ± 0.06	N/A	N/A	14	Arg45. Met46. Ala49. Ala52. Phe53. Phe56. Phe84. Asp87
ZIKV NS1 – Glycoprotein (5K6K)	*TDC_2M_ME*	-3.90 ± 0.00	3	Ser300 (3.3); Ala325 (2.8); Ly326 (3.2)	13	Pro281; Thr301; Arg324; Ala325; Lys326; Gly328; Cys329
	TDB_2M_ME	-3.77 ± 0.06	3	Ser300 (3.3); Ala325 (2.9); Ly326 (3.18)	11	Pro281; Thr301; Arg324; Lys326; Gly328; Cys329
ZIKV NS3 – Helicase Domain (5MFX)	*TDC_2M_ME*	-6.10 ± 0.00	3	Thr290 (2.7); Asp291 (3.2); His486 (3.2)	16	Phe289; Thr290; Asp291; Asp410; Ile411; Glu413; Met414; Leu430; His486
	TDB_2M_ME	-5.97 ± 0.06	3	Thr290 (2.7); Asp291 (3.2); His486 (3.3)	17	Phe289; Thr290; Asp291; Asp410; Ile411; Glu413; Met414; Leu430; His486
ZIKV NS3/NS2B – Cofactor peptide binding site (5GJ4)	*TDC_2M_ME*	-4.77 ± 0.06	2	Asn70 (3.1); Asp129 (2.8)	17	Thr68; Gly69; Asn70; Ile109; Ala127; Leu128; Asp129; Tyr130; Ser160; Tyr161
	TDB_2M_ME	-4.50 ± 0.00	1	Asn70 (3.0)	17	Thr68; Gly69; Asn70; Ala127; Leu128; Asp129; Tyr130; Ser160; Tyr161
ZIKV NS3– Protease Catalitic site (5GJ4)	*TDC_2M_ME*	-4.53 ± 0.06	1	Lys169 (3.0)	16	Leu78; Gln74; Asp122; Ile123; Ile165; Thr166; Gln167; Gly168
	TDB_2M_ME	-4.57 ± 0.12	3	Gln74 (3.0; 3.1); Lys169 (3.0)	8	Gln74; Ile123; Thr166; Gln167

ZIKV NS5 – RDRP Domain (5U04)	*TDC_2M_ME*	-5.57 ± 0.12	3	Arg623 (3.1); Asp665 (3.0); Cys667 (3.3)	15	Asn616; Val619; Gln620; Arg623; Asp665; Leu683; Lys688
	TDB_2M_ME	-5.40 ± 0.17	2	Arg623 (3.0); Cys667 (3.3)	15	Asn616; Val619; Gln620; Arg623; Asp665; Leu683; Lys688; Val689

**Virus. Protein and PDB code**	**Ligand**	**Free binding energy (Kcal/mol)**	**Hydrogen bonds**	**Residues and distance of H+ bonds (Å)**	**Hydrophobic interactions**	**Residues participating in hydrophobic interactions**
CHIKV Envelope – E2 (3N44)	*TDC_2M_ME*	-5.17 ± 0.12	1	Asn72 (2.8)	10	Arg13; Tyr15; Leu16; His29; Met70; Pro173; Thr175; Leu241; Val242; Pro243
	TDB_2M_ME	-5.17 ± 0.12	N/A	N/A	11	Arg13; Tyr15; Leu16; Met70; Asn72; His73; Pro173; Thr175; Pro176; Gln236; Val242
CHIKV Core – Protease Domain (5H23)	*TDC_2M_ME*	-4.80 ± 0.00	3	Ile144 (3.3); Phe154 (2.8); Arg156 (2.8)	17	Lys136; Thr143; Ile144; Ala150; Ala153; Phe154; Glu163
	TDB_2M_ME	-4.70 ± 0.00	N/A	N/A	15	Lys136; Val140; Gly142; Thr143; Ile144; Ala150; Leu152; Phe154; Arg156; Glu163
CHIKV NSP2 – Protease Domain (3TRK)	*TDC_2M_ME*	-5.53 ± 0.06	3	Tyr1047 (3.0); Tyr1079 (2.9); Trp1084 (3.2)	9	Ala1046; Tyr1079; Asn1082; Trp1084; Leu1205; Asp1246
	TDB_2M_ME	-5.43 ± 0.06	3	Tyr1047 (3.0); Tyr1079 (2.9); Trp1084 (3.1)	10	Ala1046; Ser1048; Tyr1079; Asn1082; Trp1084; Leu1205; Asp1246
CHIKV NSP2 – Helicase Domain (6JIM)	*TDC_2M_ME*	-5.80 ± 0.00	2	Arg172 (3.2); Thr261 (3.0)	13	Ser88; Met91; Val92; Glu95; Tyr161; His162; Ala165; Asp235; Thr261; Phe286
	TDB_2M_ME	-5.73 ± 0.06	3	Ser88 (3.3); Arg172 (3.2); Thr261 (2.7)	14	Met91; Val92; Glu95; Tyr161; His162; Ala165; Asp235; Leu238; Thr261; Phe286
CHIKV NSP3 – Macrodomain (3GPG)	*TDC_2M_ME*	-5.67 ± 0.06	N/A	N/A	20	Ala22; Asp31; Gly32; Val33; Leu108; Thr111; Gly112; Val113; Tyr142; Cys143; Trp148
	TDB_2M_ME	-5.77 ± 0.06	1	Cys34 (3.2)	22	Ala22; Asp31; Val33; Cys34; Leu108; Ser110; Thr111; Gly112; Val113; Tyr114

Virus. Protein and PDB code	Ligand	Free binding energy (Kcal/mol)	Hydrogen bonds	Residues and distance of H+ bonds (Å)	Hydrophobic interactions	Residues participating in hydrophobic interactions
Cellular Protein DDC – L-Dopa binding site (1JS3)	*TDC_2M_ME*	-7.10 ± 0.00	2	Ser149 (2.9); Lys303 (3.1)	18	Tyr79; Ile101; Phe103; Ser147; His192; His302; Phe309; Leu353; Gly354
	TDB_2M_ME	-7.07 ± 0.15	4	Ser149 (3.0; 3.1); Asn300 (3.2); Lys303 (3.0)	19	Tyr79; Phe103; Ser147; Ala148; Ser149; His192; His302; Phe309; Leu353; Gly354
Cellular Protein – α2a adrenoreceptor (6KUX)	*TDC_2M_ME*	-6.40 ± 0.00	3	Ser90 (3.2) ; Tyr109 (2.7); Tyr146 (2.9)	15	Tyr109; Asp113; Val114; Ile190; Trp387; Phe390; Phe412; Tyr416
	TDB_2M_ME	-6.00 ± 0.00	1	Ile190 (3.0)	17	Asp113; Val114; Glu189; Ile190; Trp387; Phe390; Tyr394; Phe412; Tyr416
Cellular Protein – α2b adrenoreceptor (6K41)	*TDC_2M_ME*	-6.17 ± 0.32	2	Thr97 (3.1); Ser180 (2.7)	25	Asp92; Val93; Cys96; Thr97; Ile100; Leu166; Ser180; Trp384; Phe387; Phe388; Phe412; Tyr416
	TDB_2M_ME	-5.13 ± 0.15	3	Ser69 (2.7); Tyr88 (3.0); His405 (2.9)	25	Ser69; Asn72; Glu73; Tyr88; Leu89; Cys164; Lys165; Leu166; His405; Phe408; Gln409; Phe412
Cellular Protein – β1 adrenoreceptor (7BVQ)	*TDC_2M_ME*	-6.77 ± 0.12	2	Asp1138 (3.0); Tyr1224 (3.0)	25	Asp1138; Val1139; Val1142; Phe1218; Thr1220; Tyr1224; Ala1225; Ser1228; Ser1229; Ser1232
	TDB_2M_ME	-6.60 ± 0.00	2	Ser1228 (3.0); Ser1229 (3.2)	22	Thr1135; Asp1138; Val1139; Val1142; Phe1218; Ala1225; Ser1228; Ser1229; Ser1232; Phe1340; Phe1341; Asn1344
Cellular Protein – β2 adrenoreceptor (3D4S)	*TDC_2M_ME*	-7.37 ± 0.06	4	Asp113 (2.8); Tyr199 (2.8); Ser203 (2.9); Asn312 (3.0)	20	Asp113; Val114; Val117; Phe193; Tyr199; Ala200; Ser203; Ser204; Trp286; Phe289
	TDB_2M_ME	-7.20 ± 0.00	4	Asp113 (2.9); Tyr199 (2.9); Ser203 (2.9); Asn312 (3.0)	19	Asp113; Val114; Val117; Phe193; Tyr199; Ala200; Ser203; Ser204; Trp286; Phe289; Phe290; Asn293; Tyr308; Asn312
