# Supplementary figures and images for: In Vitro and In Silico Anti-Arboviral Activities of Dihalogenated Phenolic Derivates of L-Tyrosine
Source: Molecules. 2021 Jun 5;26(11):3430. doi: 10.3390/molecules26113430 (PMC8201234; doi:10.3390/molecules26113430)

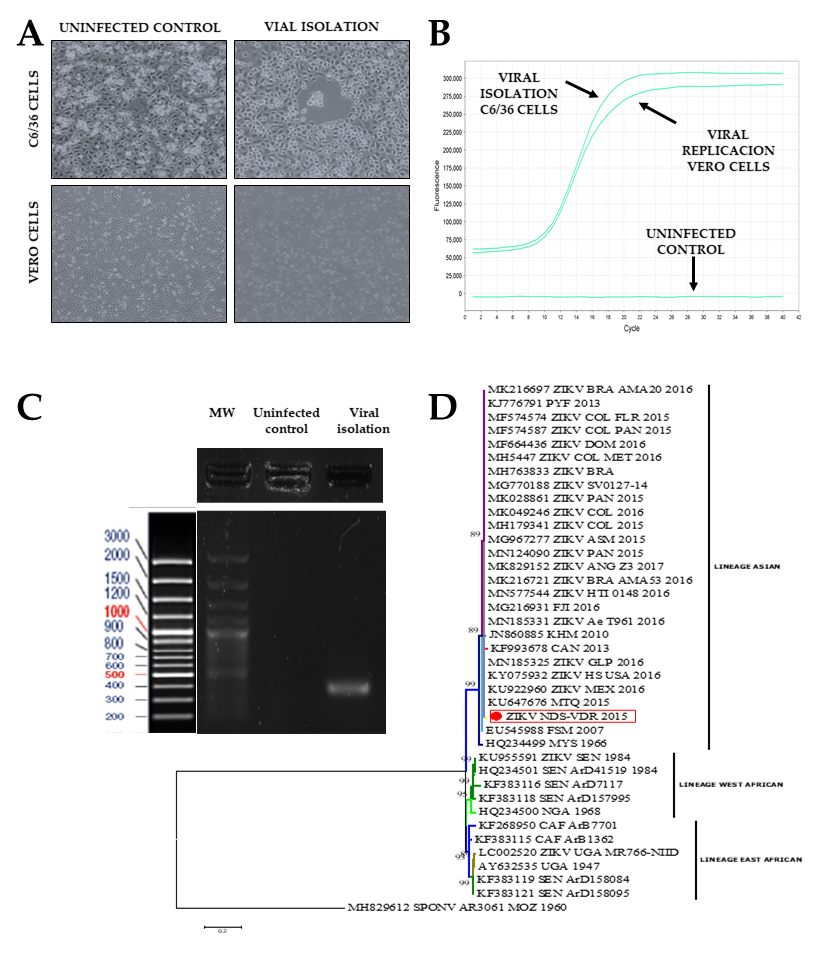

Supplement: Supplementary file 1 [file molecules-26-03430-s001.zip › Figure S2 (06-01-2021).tif]
